# Canadian Landscape Assessment of Colorectal Cancer Screening during the COVID-19 Pandemic

**DOI:** 10.3390/curroncol30100648

**Published:** 2023-10-01

**Authors:** Maria El Bizri, Malalai Wardak Hamidi, Patil Mksyartinian, Barry D. Stein

**Affiliations:** 1 Colorectal Cancer Canada, Montreal, QC H3Z 2P9, Canada; maria.bizri@hotmail.com (M.E.B.); patilm@colorectalcancercanada.com (P.M.); 2 School of Public Health Sciences, University of Waterloo, Waterloo, ON N2L 3G1, Canada; mwhamidi@uwaterloo.ca

**Keywords:** Canada, COVID-19 pandemic, oncology, colorectal cancer, cancer screening, NCCSN, Pan-Canadian, FIT, colonoscopy, delay (or interruptions)

## Abstract

The COVID-19 pandemic caused disruptions in colorectal cancer (CRC) care by interrupting CRC screening across Canada, posing problems for program participants, patients, and physicians and no clear understanding of how provincial healthcare systems would adapt in the face of another pandemic or shock to the system. A nationwide online survey targeted to members of the National Colorectal Cancer Screening Network (NCCSN) using the SurveyMonkey platform was conducted to gain insight into the impact of the pandemic on CRC screening from March 2020 to March 2022 across all thirteen Canadian jurisdictions. The survey included 25 multiple-choice and free-text questions. Both quantitative and qualitative methods were used to analyze the data using Microsoft Excel and NVivo software. Twenty-one provincial and territorial representatives participated in the survey conducted between 13 May 2022 and 27 October 2022. All jurisdictions (100%) reported decreased screenings, including fecal immunochemical testing (FIT) or Fecal Occult Blood testing (FOBT) procedures, and subsequent diagnostic colonoscopies. The average wait time for colonoscopies due to a positive FIT/FOBT was 76 days. To mitigate the backlog and initiate an effective intervention plan, representatives highlighted some key points, including the importance of prioritizing high-risk patients. Survey results concluded that the COVID-19 pandemic impacted CRC screening across Canada. This landscape assessment can help inform intervention measures and policy-related solutions to create greater resilience for CRC screening in provincial and territorial healthcare systems.

## 1. Introduction

Colorectal cancer (CRC) is the fourth leading cancer diagnosed in Canada and the second most common cause of death among men and third among women in 2022 [1]. In 2022, there were approximately 24,300 Canadians diagnosed with colorectal cancer and approximately 9400 died from the disease [1]. Over the years, the Canadian healthcare system has reported declines in the incidence of CRC; yet it still accounted for 10% of all cancer-related mortalities with only a 67% five-year survival rate [2]. The Canadian healthcare system has always prioritized colonoscopies and regular screenings to help detect CRC in its earliest, most curable stages. Healthcare institutions use endoscopic techniques, such as flexible sigmoidoscopy and colonoscopy or stool-based diagnostics, per established guidelines. All provinces and territories recommend screening asymptomatic individuals at average risk of developing CRC between the ages of 50 and 74 or 75 every 12–30 months with a fecal test (FT), either the guaiac fecal test (Ft) or fecal immunochemical test (FIT) [3]. At the time of the survey, all provinces and territories had organized CRC screening programs with the exception of Quebec and Nunavut, where screening was opportunistic. 

Unfortunately, the global spread of the COVID-19 pandemic has had significant consequences for preventive cancer screening care, including the cessation of CRC services in all provinces and territories in Canada [4,5]. Hospitals across Canadian provinces considered the postponement of elective surgical or medical procedures, including colonoscopies, in response to the concerns that elective procedures may expose hospitalized patients to the SARS-CoV-2 virus [6,7]. The notable decrease in the number of individuals screened for cancer was likely due to a combination of factors, including fear of exposure to the virus, concerns about overburdening the healthcare system, and changes in the way cancer screening services were delivered [8]. Both low participant turnout in FIT programs and inconsistent referrals of individuals who tested positive for the FIT procedures to the subsequent diagnostic colonoscopies contributed to the underperformance of screening initiatives in the country [9]. 

Most screening programs were interrupted, and there was approximately a 30% decrease in new patient referrals in Saskatchewan, Ontario, and Quebec, and 50–75% of treatments continued by using virtual tools such as phone calls [10]. In Quebec, by way of example, there was a reduced performance of screening initiatives due to both low participant turnout in FIT programs and inconsistent referrals of patients who tested positive for the FIT and subsequent diagnostic colonoscopies [11]. The cancellation of scheduled colonoscopy appointments and higher rescheduling rates further disrupted screening programs, with many Quebec residents cancelling their appointments due to the fear of COVID-19 [12]. Importantly, these reductions were not unique to Quebec alone, as other provinces across Canada and countries in Europe experienced similar patterns of CRC screening interruptions [12].

The decrease in CRC screening and diagnostic testing services due to the pandemic raises concerns over the near-term and long-term status of cancer prevention and treatment. In fact, after observing recent trends in cancer progression, Lui et al. estimated that if the healthcare system fails to contain the ongoing reductions in diagnostic procedures, then 6.4% of CRC cases would exhibit greater stage shifting [13]. Stage shifting due to delays in diagnostics would result in upstaging as more patients would progress from stage I to stage IV carcinomas [13]. Moreover, another 20% decrease in endoscopy services from the current levels would still trigger a further 7.2% increase in upstaging of the carcinomas [13]. New cases of CRC in Canada could rise by 0.6% and fatalities by 0.8%, assuming that there is a commensurate increase in diagnostic interruptions over a year [14].

Moreover, stage shifting could lead to a poorer prognosis, as the stage of a carcinoma is a significant prognostic factor for CRC [15]. Consequently, individuals with a higher stage of CRC, such as Stage IV, have a poorer prognosis and decreased chance of survival [15,16].The lack of preparedness and planning in delivering primary care services both before and during the pandemic led to further disruption and reduction in cancer screening. Earlier access to urgent catch-up screenings by screening and diagnostic institutions could mitigate the forecasted adverse outcomes or even eliminate them [4]. The impact of the pandemic on CRC cancer screening delays in Canada [4,9] and across specific provinces (e.g., Ontario) [8] has been reported in a few articles [4,8,9,17], but further research is needed to investigate the impact of COVID-19 on provincial and territorial CRC screening programs.

### Objective

The aim of the current study is to better understand the impact of COVID-19 on provincial and territorial CRC screening programs with the goal of exploring solutions to mitigate the impact of missed screenings, increase screening rates and create greater resilience in CRC cancer prevention and screening in Canada. The results will help provide tangible information that will be shared with the National Colorectal Cancer Screening Network (NCCSN) for further analysis and feedback with the aim of further collaboration, development, and adoption of best practices.

## 2. Materials and Methods

### 2.1. Target Population

The target population of this survey included representatives of the Canadian provincial and territorial CRC screening programs who are all members of the NCCSN. 

### 2.2. Survey Design and Development

A 25-item survey (see Appendix A) of multiple choice and free-text responses was created using SurveyMonkey to understand the impact of the COVID-19 pandemic on CRC services in Canada between March 2020 to March 2022. The questions were designed internally by the Colorectal Cancer Canada (CCC) team and reviewed and edited by a third-party scientist as well as members of the Canadian Partnership Against Cancer (CPAC). The questions were available in English and covered four themes: interruptions, quantifiable effects, intervention plans, and recommendations. The first question was a mandatory consent question, which was followed by three questions on the participant’s personal and demographic information, such as name, email address, and province or territory represented. The following three questions (two context questions and one follow-up question) probed into the possible occurrence of COVID-related interruptions of CRC screening services such as fecal immunochemical testing (FIT) or fecal occult blood test (FOBT) procedures and subsequent diagnostic colonoscopies across provinces and territories. The next five questions inquired about the quantifiable impacts of CRC screening interruptions—either regionally (Q8), on the case basis of delays in colonoscopies (Q9–11), or temporal changes in all the screening services (Q12). Questions 13 to 18 (Q13–18) inquired about the existence of an intervention plan addressing the disruptions that the pandemic had on FIT/FOBT and colonoscopies. The remaining six questions (Q19–24) targeted the respondents’ opinions on the most appropriate solutions for mitigating the effects of the pandemic on colorectal screening services. 

### 2.3. Survey Administration and Data Management

#### 2.3.1. Survey Administration

The survey was disseminated to the NCCSN by CPAC on behalf of CCC. The survey was administered using SurveyMonkey’s online platform. In the email, participants were informed about the objective of the project and were given the option to participate in the survey. The survey was disseminated via email on 13 May 2022, which was followed by weekly follow-up emails and one phone call until all NCCSN members of provincial and territorial screening programs were represented. 

The first four questions (consent and demographic questions) were mandatory to answer, while the subsequent questions were all optional to answer. Respondents had the ability to change their responses using the “return” button at the bottom of the screen and were also able to complete the survey at a later point without losing their previous responses. The data collection period started on 13 May 2022 and ended on 27 October 2022.

#### 2.3.2. Analytic Methods

Data were extracted and collected from SurveyMonkey in Excel format (csv file). Data were analyzed using Microsoft Excel to descriptively analyze the results by calculating the measures of central tendency (e.g., mean, median, mode) and variability (e.g., standard deviation and variance). Categorical variables were summarized using frequency tables, figures, and charts created in Excel, which graphically depict the data trends, such as the number (n) and percent (%) of provinces/territories out of the total number of provinces/territories (N). Accordingly, the numerical responses were aggregated to understand the common trends among the sample and examine the quantitative portion of the dataset.

Moreover, to assess the qualitative portion of the dataset, free-text responses from the survey were transferred to a Word document and were analyzed using NVivo (qualitative data analysis software Version 12) (Lumivero, Denver, CO, USA). Specifically, a thematic analysis approach was utilized using the framework established by Braun and Clarke to identify the latent themes and patterns within the data, and an inductive method was used to allow for the data to identify the key themes.

#### 2.3.3. Data Management 

Incomplete responses were excluded from the final data before the analysis was conducted. When possible, one submission per jurisdiction was requested from participants. In case of duplicate responses (same province/territory answered by different representatives), and if faced with conflicting answers from respondents of the same jurisdiction, responder agreement was analyzed, and the majority rule was applied. No reconciliation was performed on conflicting data from multiple participants of the same jurisdiction, and no data imputation was performed. The survey data were kept on a secure server in the survey platform “SurveyMonkey”, which is compliant with Health Canada’s standards for data security.

#### 2.3.4. Data Validation

In order to verify the accuracy of the data and information collected, the preliminary results section, which included analyses and graphs, was shared with representatives on 9 March 2023, through email, with a subsequent follow-up email sent on 16 March 2023. Representatives were given a deadline to review and validate the information by 20 March 2023; however, as requested by one of the representatives, an extension was granted until 22 March 2023, whose response was received by 21 March 2023. All provinces/territories (P/T) representatives were given the opportunity to validate their results, and out of the 15 P/T representatives, six provinces/territories: Quebec, British Columbia, Nova Scotia, Newfoundland and Labrador, Prince Edward Island, and the Northwest Territories provided additional feedback. Legitimate concerns were addressed, and corrections were made if necessary.

## 3. Results 

### 3.1. Representative Demographics

A total of 21 provincial and territorial representatives participated in the survey, with 15 out of 21 participants (71%) completing the survey between 13 May 2022, and 27 October 2022. When possible, only one submission per province and territory (P/T) was asked for; however, there were duplicate responses from some provinces, including Ontario, Alberta, and the Northwest Territories. The number of representatives from each P/T is shown in Table 1.

### 3.2. Impact of COVID-19 on Colorectal Cancer Screening Services

**Figure 1:**
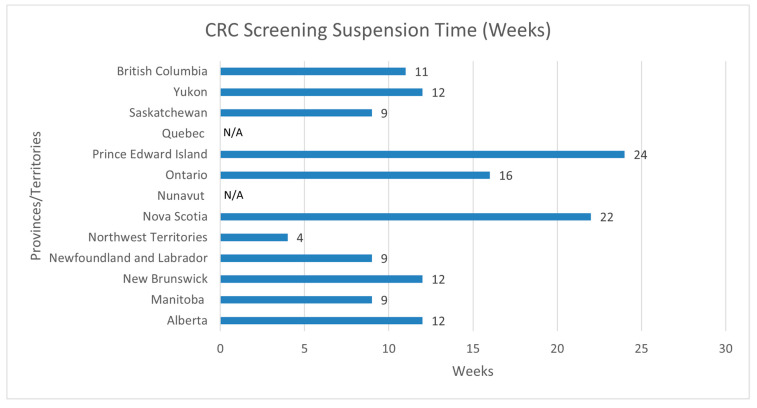
CRC screening suspension time (weeks) for FIT and colonoscopies. Note. Nova Scotia specified the time of interruptions (weeks) for colonoscopy and FIT, which was 12 weeks for endoscopy services from mid-March 2020 to late May 2020 and 32 weeks for FIT from 9 March to 15 October 2020. Prince Edward Island noted that one of the factors that influenced the timing of reinstatement was the reinstatement of non-urgent endoscopy services, which restarted in June/July of 2020, and by the end of 2020, the backlog of endoscopy services was cleared. This played a significant role in the approval required for reinstating the FIT screening plan. Additionally, the deployment of staff supporting program work was also an influencing factor in the timing of reinstatement. The remaining provinces and territories did not provide specific time interruptions for each CRC screening service.

Eleven of the thirteen jurisdictions (85%) reported interruptions in their CRC screening services, including fecal immunochemical testing (FIT) procedures and the subsequent diagnostic colonoscopies (Figure 1). Quebec and Nunavut, two jurisdictions with no established CRC screening programs at the time of this article, but who are in the process of implementing a program, reported reductions in screening for those who did not see their healthcare practitioner during the first wave of the COVID-19 pandemic. The duration of interruptions varied per jurisdiction, as PEI reported the longest period of suspension (24 weeks), while the Northwest Territories reported the shortest suspension period (4 weeks). CRC screening services were suspended for an average of 13 weeks across the eleven provinces that reported suspensions, while the most common was 12 weeks—as three out of the thirteen jurisdictions reported suspending services for 12 weeks (AB, NB, YT).

### 3.3. Quantifiable Impacts of the Interruption 

The impact of CRC screening services’ suspension was reported to be different in urban and rural areas of Canadian jurisdictions. Five out of eleven jurisdictions (45.5%) (MB, ON, PEI, SK, and BC) reported that both rural and urban areas were impacted equally. At the same time, three out of eleven jurisdictions (27.3%) (AB, NB, and QC) reported that the effect depended on the impact of COVID-19 within the various jurisdictions. Two out of eleven jurisdictions (18.2%) (NL and NS) reported that urban areas in their provinces were more impacted by the suspensions. In contrast, the Northwest Territories was the only jurisdiction that reported rural areas to be more impacted by the suspension of the screenings (9%).

### 3.4. Missed Colorectal Cancer Screenings 

The representatives were requested to provide an approximate number of missed CRC screenings (this includes reduced volume) or year-over-year changes. All jurisdictions (100%) reported a decrease in the number of screenings performed. Newfoundland and Labrador reported 4000 fewer FITs between March 2020 and March 2022. Alberta reported a decrease in both FIT (293,000 to 207,000) and colonoscopies (113,200 to 87,500) between 2019 and 2020—representing a 30% decrease for FIT and 25% for colonoscopies. In Ontario, for the average risk CRC patients, the volume of FIT declined by 4.4% in March 2020 and it declined by 78.3% in April, 90.3% in May, and 91.3% in June; however, afterward, the volume began to recover as FIT kit mailing resumed [8]. Furthermore, in Ontario, the range (33.2–52.8%) of colonoscopies performed was lower from February to April 2020, with the lowest month being March 2020 (9.2–12.8%) [8]. Quebec reported 166,000 fewer FIT analyses (647,000 to 481,000) and 60,000 fewer colonoscopies (267,000 to 207,000) between April 2020 and March 2021—representing a 26% decrease for FIT and 22% decrease for colonoscopies.

### 3.5. Awaiting Colonoscopy due to Positive FIT or Other Reasons

Representatives were asked to provide information on the number of people currently awaiting colonoscopies or endoscopies due to a positive FIT or for other reasons (from March 2020 to March 2022). From the available data, Saskatchewan reported that the average delay for colonoscopies due to a positive FIT was 131 days, Quebec reported 4100 individuals were waiting to have a colonoscopy, 2300 of which were on target being within 60 days of a positive FIT, and 1800 were over the said delay. Quebec reported that approximately 121,000 colonoscopy requests were pending as of 15 April 2022, (including primary and follow-up for all reasons). New Brunswick reported that 24 FIT-positive participants were waiting to be booked for a colonoscopy as of 6 June 2022. The remaining provinces and territories were unable to provide information for this question.

**Figure 2:**
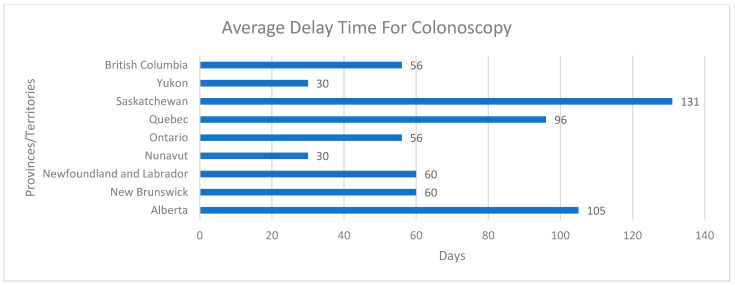
Average delay time (in days) for colonoscopies (due to positive FIT) in different provinces/territories.

Representatives reported the average delay times for colonoscopies due to a positive FIT between March 2020 and March 2022 (Figure 2). Saskatchewan reported the highest average delay time of 131 days. Quebec had an average delay time of 96 days, and Nunavut had the lowest delay time of 30 days. The most commonly reported delay time was 56 days (ON and BC) and 60 days (NFL and NB).

### 3.6. Addressing the Backlog of CRC Screening and Procedures 

**Figure 3:**
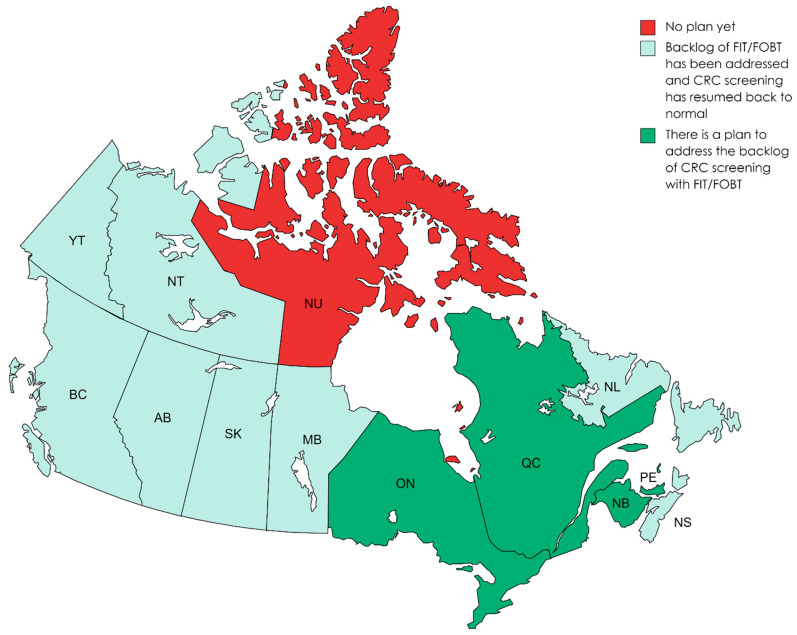
Addressing the backlog of FIT/FOBT (between March 2020 to March 2022). Note. Newfoundland and Labrador quickly caught up on their backlog of FIT, as they had a coordinated approach with regional health authorities and prioritized FIT based on colonoscopy capacity. Accordingly, they had a successful recovery strategy for addressing their backlog, which they plan to reuse for COVID and future obstacles. Nova Scotia noted that they did not resume FIT mailings until mid-October 2020, as they waited until the backlog of FIT-positive colonoscopies was addressed before resuming FIT mailings.

**Figure 4:**
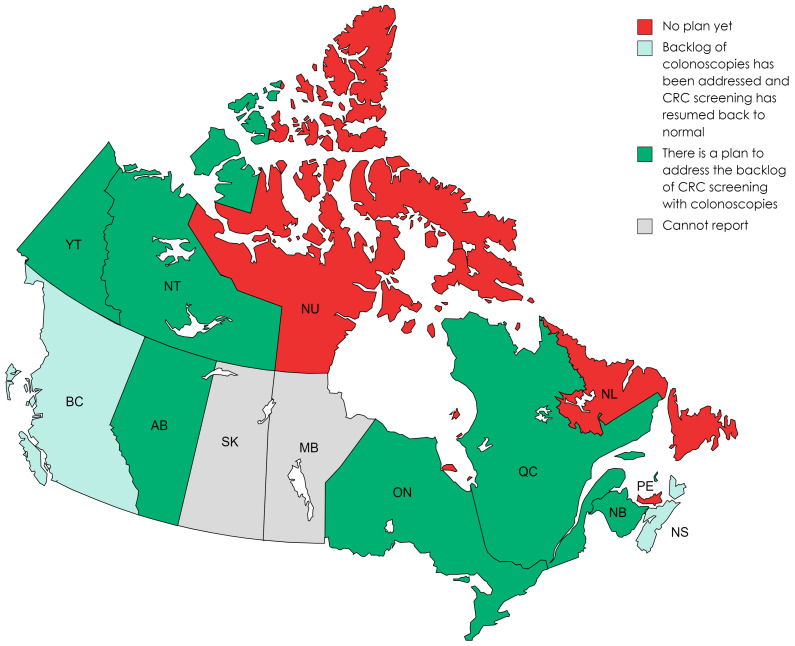
Addressing the backlog of colonoscopies (March 2020 to March 2022). Note. Newfoundland and Labrador did have a plan to catch up on colonoscopies based on FIT-positive data and procedures that could be accessed in each endoscopy unit. There was a brief period of reduced service for FIT-positive colonoscopies, but when the system “re-opened”, there was immediate consensus among the regional health authorities, which was based on their historical data on the importance of FIT-positive colonoscopies and the need for these procedures to continue. Meanwhile, Nova Scotia noted that when endoscopy services resumed in late-May 2020, there was a backlog of FIT-positive colonoscopies, but the backlog was addressed by mid-October 2020.

Representatives were asked how they plan to address the backlog of CRC screening with FIT tests and colonoscopies (Figure 3 and Figure 4). Although 61.5% of the respondents mentioned that the backlog for FIT had already been addressed and screening had resumed back to pre-pandemic levels, the remaining 30.8% reported that they had a plan to address the backlog of FIT. Nunavut reported that there was no plan as of yet to address the backlog of FIT. 

While British Columbia and Nova Scotia reported that the backlog of colonoscopies was addressed, 46% of the respondents reported they had a plan to address the backlog. However, 23.1% of the respondents reported that they had no plan to address the backlog of colonoscopies, and Saskatchewan and Manitoba did not report how they were addressing the backlog. 

The main sub-themes that emerged from the jurisdictions’ responses on their plan to address CRC screening backlogs included prioritization and targeting high-risk patients, intersectoral collaboration and communication, CRC screening awareness campaigns, financial support to increase capacity, and future research considerations.

#### 3.6.1. Prioritization (Targeting High-Risk Patients)

Representatives highlighted the importance of prioritizing high-risk patients (e.g., those with a positive FIT result) to address the backlog of CRC screening and other procedures. The majority of participants discussed how patients who have a positive FIT should be prioritized for follow-up testing with colonoscopy before average-risk primary colonoscopy screening. Ontario mentioned they utilized a prioritization framework from Cancer Care Ontario to help endoscopists prioritize cases. In accordance with the framework, GI endoscopy cases were assigned to one of the five priority levels based on a risk assessment of morbidity and mortality outcomes [18]. Patients with abnormal FIT results were assigned to the second highest priority level, while those who are at average risk for CRC (e.g., could be screened with a FIT) were assigned to the lowest priority level. Similarly, other jurisdictions (AB, PEI, and NU) noted that they also implemented case prioritization protocols and targeted screening initiatives for participants who were overdue for CRC screening and at higher risk (e.g., positive FIT). For instance, Quebec utilized a prioritization framework integrated into their colonoscopy request form with five priority levels based on a risk assessment of morbidity and mortality outcomes. Lastly, Quebec also provided formal instructions to prioritize endoscopies for patients with positive FIT results and planned to make service corridors to the near endoscopy establishments to ensure patients received their colonoscopies on time. 

#### 3.6.2. Intersectoral Collaboration and Communication

Representatives emphasized the importance of intersectoral collaboration and communication with different stakeholders to address the backlog of CRC screening tests and other procedures. Various jurisdictions noted that they regularly communicated and engaged with their partners within the healthcare system to coordinate CRC screening services and reduce booking and wait times for patients. For example, Quebec noted that their program frequently communicated with individual colonoscopy sites to focus on addressing booking and wait time issues and implemented a centralized method to offer patients colonoscopies at different locations, which was contingent on wait times and availability. Ontario noted they were involved in continuous engagement with regional partners to encourage CRC screening in primary care and other healthcare settings.

#### 3.6.3. CRC Screening Awareness Campaigns 

Awareness campaigns were noted by various representatives as a way to mitigate the impact of COVID-19 on the backlog of CRC screening and follow-up volumes. Awareness campaigns mentioned by the respondents consist of screening invitations from organized cancer screening programs (e.g., Cancer Care Ontario) and primary care providers (PCPs) as well as health promotion campaigns to encourage screening among the eligible population (e.g., adults aged 50 to 74). Quebec mentioned that they had planned awareness campaigns for PCPs to encourage discussions on screening with their patients who may have missed a screening cycle during the COVID-19 pandemic. Similarly, other jurisdictions (ON, NS, and NB) stated they developed CRC screening invitations and awareness campaigns (e.g., social media campaigns and toolkits for PCPs) to increase screening participation and the coverage rate among the eligible screening population.

#### 3.6.4. Financial Support

Financial support was also cited as an important factor for addressing the backlog, as most representatives noted that funds were necessary to expand the capacity for CRC screening services and procedures. Yukon noted that funding from the Canadian Partnership Against Cancer (CPAC) in 2021 helped improve the turnaround time for patients receiving a positive FIT result to undergo a follow-up colonoscopy. Quebec also noted that additional funding was made available to fund additional human resources and increase the capacity for colonoscopies beyond the previous baseline year 2019.

#### 3.6.5. Future Research Considerations 

Future research considerations were noted by various jurisdictions in the context of the backlog of CRC screening procedures and the potential increases in later-stage CRC diagnoses. Alberta noted that the incidence of CRC cases is predicted to increase considerably this year, but this challenge will be addressed by their COVID-19 recovery plan. While Northwest Territories are planning to review and amend their CRC screening guidelines, PEI is working on updating the number of CRC cases diagnosed throughout the pandemic, and British Columbia has recently updated their screening guidelines. 

### 3.7. Increasing Uptake in Screening Programs

Representatives were asked if there would be any changes to their CRC screening program invitations to increase program participation uptake. Most jurisdictions (*n* = 8) mentioned that they have already implemented changes or are planning to implement changes in the future to increase program uptake. Meanwhile, Manitoba, Newfoundland and Labrador, and Nova Scotia (*n* = 3) mentioned either no changes are necessary or that changes cannot be made until the capacity for colonoscopies is increased. The main sub-themes that emerged from this question included FIT distribution and awareness/educational campaigns.

#### 3.7.1. FIT Distribution

Representatives of the jurisdictions highlighted the significance of their FIT distribution methods, such as mailing FIT kits to increase program uptake. Various jurisdictions noted that they have implemented or are considering mailing FIT kits directly to individuals to meet the CRC screening demands. Prince Edward Island noted that they piloted a direct mail approach, where FIT can be returned via mail instead of in-person drop-offs. Northwest Territories continued the planned expansion of their organized screening program, which included mailing FITs directly to patients for them to complete and drop off at a lab or health center. Ontario reported that they worked with laboratory partners to grow the number of FIT mailing kits that can be sent out to eligible participants to meet the demand.

#### 3.7.2. Awareness/Educational Campaigns (for CRC Screening)

Representatives also noted that they are planning to utilize awareness and educational campaigns to increase program participation. Awareness campaigns include initiatives such as marketing campaigns and invitation letters to increase participation in the CRC screening programs, while educational initiatives include virtual sessions for learning about pre-colonoscopy procedures. Ontario mentioned that they developed a flyer to include screening invitation letters to deliver information about the safety of screening and the potential harm related to delaying screening due to COVID-19 as well as creating cancer screening promotion campaigns via social media to encourage screening uptake among the eligible screening population.

### 3.8. Addressing Overdue FIT and Colonoscopies

**Figure 5:**
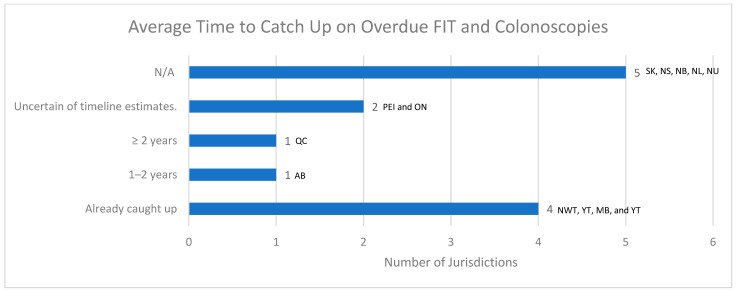
Average time to catch up on overdue FIT and colonoscopies.

Representatives were asked how long it would take to catch up on overdue FIT and colonoscopies (Figure 5). Four out of thirteen jurisdictions (31%) reported that they were already caught up on overdue FIT and/or colonoscopies based on expected volumes between March 2020 and March 2022. Meanwhile, both Prince Edward Island and Ontario reported uncertainty about the exact timeline. Quebec reported that it would take two years or longer to catch up on colonoscopies, while Alberta reported that it would take 1 to 2 years to catch up on colonoscopies. Information from the remaining jurisdictions was unavailable.

#### Reaching Pre-Pandemic System Performance

Ten out of thirteen jurisdictions (77%) have already reached their pre-pandemic system performance for FIT and/or colonoscopies. Alberta reported having reached pre-pandemic system performance for “both FIT and colonoscopy”. Manitoba reported that its FOBT distribution within the province had also reached its pre-pandemic performance. Northwest Territories expect their FIT screening to continue to increase as their program expands, and their colonoscopy levels are “roughly similar to pre-pandemic performance”. Ontario reported that they had caught up and exceeded pre-pandemic performance for FIT per month in 2022, while the number of hospital-based colonoscopies completed per month in 2022 was still slightly below pre-pandemic levels. Quebec reported that with assistance from the private sector and public networks, they expect that the “colonoscopy situation will be resolved within two years to reach the pre-pandemic level”, but due to human resource challenges, the objective of achieving pre-pandemic levels could be longer than the estimated two years. The Yukon reported that they reached pre-pandemic numbers by September 2021, while Nova Scotia reported that they only suspended endoscopy services for less than 8 weeks from mid-March 2020 until May 2020, and FIT mailings for 7.5 months—after which pre-pandemic levels were achieved.

### 3.9. The Expectation of Higher Positivity Rate and the Need for More Colonoscopies 

Representatives were asked, “Given the fact that there has been a 2-year hiatus, does your program plan to simply resume screening as before with the expectation of a higher positivity rate and a need for more colonoscopies?”

The Northwest Territories, Quebec, and Prince Edward Island reported that they expect an increased positivity rate and/or a necessity for more colonoscopies. For example, the Northwest Territories noted that they are continuing to roll out their new FIT screening program and expect higher-than-average positivity rates for CRC. Seven out of thirteen jurisdictions (61.5%) (AB, MB, NB, NL, ON, BC, and NS) reported already being back to their pre-pandemic levels. No data are available to report for the remaining three jurisdictions (NU, YT, SK).

### 3.10. Mitigating Future CRC Screening Interruptions 

**Figure 6:**
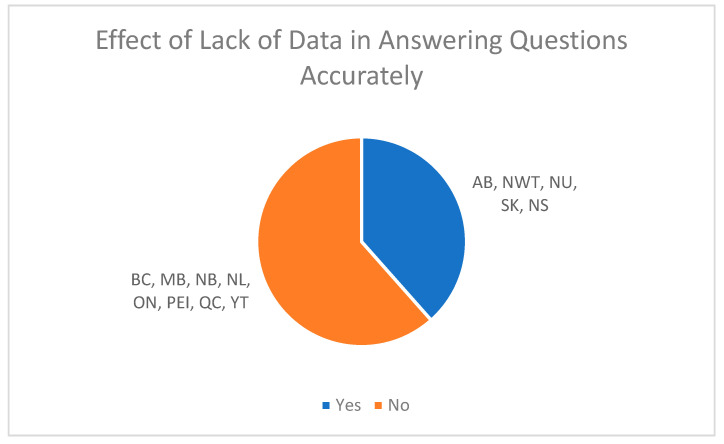
Responses on the effect of lack of data in answering questions accurately.

Representatives were asked to provide their opinions about which intervention strategies would have the greatest impact in avoiding future interruptions in CRC screening (FIT) and follow-up colonoscopies. Regarding FIT, the majority of jurisdictions noted that (1) increasing resources for community health centers to distribute FIT kits (38%) and (2) making FIT more readily available (31%) would be the best approaches for mitigating future interruptions (Figure 6). For follow-up colonoscopies, the majority of jurisdictions noted that (1) increasing human resources in hospitals (54%) and increasing endoscopy service hours (38%) would be the best approaches for mitigating future interruptions. The main sub-themes that emerged from this question included better planning/strategic planning and awareness initiatives.

#### 3.10.1. Better Planning/Strategic Planning

Representatives highlighted the importance of better strategic planning to mitigate future interruptions in CRC screening (FIT) and follow-up colonoscopies. Various jurisdictions mentioned that better planning regarding booking systems, contingency plans, human resources, and infection control would be necessary to implement in the future. The Northwest Territories noted that providing centralized staffing dedicated to territorial screening would help safeguard their CRC screening program. Saskatchewan noted that standardized booking systems and pathways would grow efficacy and increase accessibility. Quebec reported that during the COVID-19 pandemic, recommendations based on essential activities that correspond to provincial alert levels were developed, and accordingly, FIT-positive requests were included as a clinical indicator that was prioritized among other clinical indicators.

#### 3.10.2. Awareness Initiatives

Representatives mentioned that raising awareness through public campaigns and mailing FIT kits could help mitigate future CRC screening interruptions. Prince Edward Island mentioned that public messaging is important for promoting support and developing confidence for CRC screening, including messaging highlighting the appropriateness and importance of CRC screening even during a pandemic. 

### 3.11. Additional Data and Types of Data for Mitigating Future CRC Screening Interruptions 

Representatives were asked whether additional data would have helped them mitigate the interruptions in FIT screening and endoscopy. While 23% of representatives were uncertain as to whether additional data would have helped, the remaining representatives said “no” to the aforementioned question. Alberta noted that data collection was impacted, and they did not have “good data” to assess the actual impact of the COVID-19 pandemic on FIT screening and endoscopy interruptions. Similarly, the Northwest Territories mentioned that they had minimal CRC-specific data before the COVID-19 pandemic and have started to generate and track data for CRC screening programs.

The screening program representatives were also asked to provide their opinions about what types of data would have helped them mitigate the interruptions in FIT screening and endoscopy. Out of the four representatives who responded, Northwest Territories and Prince Edward Island noted that data for assisting and guiding the triage of CRC screening services would be useful for mitigating interruptions. Northwest Territories mentioned that data on how to most appropriately re-triage delayed patients may have been valuable, such as data that might help to prioritize individuals with positive FIT when individuals were beyond the recommended maximum wait times for colonoscopy follow-up. Moreover, New Brunswick noted advanced information on booking delays, and Alberta reported that continuous collection and reporting are important data types/collection methods. Meanwhile, Prince Edward Island noted that data on full referrals and waitlists for endoscopy services for assisting triage is important to consider.

### 3.12. Other Solutions for Mitigating Future CRC Screening Interruptions

Representatives were asked to suggest other solutions for mitigating the interruptions in FIT screening and endoscopy. Four representatives responded to this question. New Brunswick and the Northwest Territories noted that increased resources for endoscopies and resource support for data collection were necessary to address this issue. New Brunswick also indicated that increasing resources for CRC screening and endoscopy capacity in the healthcare system are important solutions to consider. Prince Edward Island and Ontario also noted that a navigation program for CRC screening, open discussions, up-to-date data, and strategic planning are other solutions that should be considered.

### 3.13. Lack of Data Collection on Answering Questions Accurately

Finally, representatives were asked whether the lack of data collection on CRC screening, diagnostic, and other related procedures prevented them from answering the survey questions accurately (see Figure 6). The majority of representatives (62%) responded “no”, that the lack of data collection factors had not impacted their ability to accurately respond to the survey questions, while the remaining representatives (38%) responded “yes” to the aforementioned factors.

## 4. Discussion

CRC screening programs are implemented at the provincial and territorial (P/T) level with the healthcare system managed and administered through a centralized organizational structure established and financed by the P/T governments. Currently, organized CRC screening programs exist in nine provinces and two territories, while in Nunavut and Quebec, CRC screening is opportunistically ordered by healthcare providers (HCPs) [19]. CRC screening programs vary by jurisdiction, with some using automated mail-in fecal immunochemical testing (FITs) and others relying on in-person appointments with their PCPs to have access to FIT and colonoscopy services. The aim of this study is to explore the effects of the COVID-19 pandemic on CRC screening services in Canadian jurisdictions and study the steps taken by them to mitigate the effects.

Efforts were made throughout the pandemic to slow down the spread of COVID-19 through various public health initiatives. While these efforts were helpful in slowing down the spread of COVID-19, they resulted in the disruption of critical healthcare services including P/T CRC screening programs. 

### 4.1. Main Findings

During the time period of March 2020 to March 2022, there was a reported decrease in the number of CRC screening services including both FIT and colonoscopies performed in all jurisdictions across Canada. Because the pandemic reduced the availability of healthcare personnel and the movement of people, there was a decline in access to CRC screening services. While the pandemic was unexpected, the interruption of CRC screening services indicated a lack of adequate preparedness and resilience in our healthcare systems. 

Individuals were reluctant to seek out CRC screening services due to the fear of being infected with COVID-19, and this contributed to missed CRC screenings [20]. In addition, there are other psychological factors that can negatively influence an individual’s decision to adhere to cancer screening, such as fear of a positive test, lack of understanding of the procedure, anticipation of pain, anxiety, and sentiments of embarrassment and vulnerability [21]. A study by Lee et al. (2022) identified health inequalities in CRC screening programs in rural and urban areas in Canada as additional reasons for a decline in CRC screening [20]. Similarly, the survey results indicated that some of the P/Ts experienced disparities in rural areas, such as in the Northwest Territories, while Newfoundland, Labrador, and Nunavut reported that urban areas were more impacted. The remaining jurisdictions reported that both urban and rural areas were equally impacted by the suspensions of screening services.

By according priority to high-risk populations and instituting adaptable screening programs, healthcare providers and organizations can bolster the resilience of CRC screening initiatives, rendering them more capable of weathering disruptions stemming from pandemics or other public health crises. Guidelines for the prioritization of individuals at higher risk of CRC can help to identify high-risk populations, using age, family history of CRC, or certain medical conditions.

A more targeted approach to screening could help allocate scarce resources when and where they are most needed during a pandemic and help ensure that those most in need of screening would be able to receive it in a timely manner even in the face of disruptions caused by the pandemic.

Respondents to the survey highlighted the significance of flexibility and adaptability of cancer screening programs, to quickly adapt and make changes in the delivery of healthcare services in order to mitigate CRC morbidity and mortality. Shifting to mailing out FIT kits directly to participants in the screening program rather than the individual participant having to visit a healthcare professional first to obtain the kit is an example of the flexibility and adaptability highlighted to respond to the pandemic. 

The pandemic’s impact on CRC screening can be significantly reduced by the enhanced availability of information and resources in the healthcare system (such as FIT-positive data). Increasing resources in the healthcare system, such as providing additional funding for CRC screening programs or deploying healthcare personnel to support screening services in underserved areas, may have helped to ensure these services remain accessible and available during the COVID-19 pandemic. For instance, to address the behavioral barriers associated with CRC screening (e.g., fear, anxiety, etc.), it is important to have patient navigators and community health liaisons who can promote CRC screening adherence by influencing patients’ awareness and knowledge of colonoscopies [19].

Healthcare practitioners and organizations might have efficiently identified and managed individuals at the highest risk of having CRC if they had access to the necessary data on how to prioritize and re-triage delayed patients. The use of risk stratification techniques could have assisted in identifying and prioritizing people who are most likely to have developed CRC during the CRC screening suspensions and interruptions. Furthermore, based on an individual’s particular risk factors and clinical history, healthcare providers could have created strategies for follow-up screening. The availability of this data could have aided in mitigating the pandemic’s impact on CRC screening programs and mitigating the negative consequences of screening delays.

### 4.2. Comparison with Other Studies

Several other studies have examined the effect of the pandemic on cancer screening services in some Canadian P/Ts. In the study by Walker et al. (2021), the researchers looked at the impact of COVID-19 on pan-cancer screening rates in Ontario [8]. The study found that the effects of disruptions from the pandemic were more severe at the early stages of the pandemic [8]. For example, in Ontario, the reduction in breast, cervical, and colorectal cancer screening programs was reported to be over 41% (cumulatively) between 2019 and 2020, representing 951,000 fewer screening tests delivered [8]. Similarly, our results indicated that all jurisdictions across Canada reported a decrease in the number of CRC screenings performed between March 2020 and March 2022.

Croswell et al. (2021) looked at how cancer screening services were impacted by the pandemic in the United States and noted a significant decrease in the number of cancer screenings completed during the pandemic [22]. Croswell et al. (2021) also had similar findings on the strategies for mitigating the effects of the pandemic on screening services, in which prioritizing health services and high-risk patients were some ways to develop resilience to the pandemic [22]. Our results also found that prioritizing high-risk patients was significant for prevention and addressing the backlog of CRC screening as well as building healthcare system resilience. 

### 4.3. Strengths and Limitations

Our study has several strengths, one of which is that a pan-Canadian sample selection was utilized, ensuring that the findings are representative of the P/Ts across Canada, which supports the generalizability of the study. Furthermore, the study’s findings can be used to educate future decision-makers, helping healthcare practitioners and governments prioritize CRC screening services, build mechanisms for future interruptions or shocks to the healthcare system, and improve the resilience of CRC screening programs.

While the current study contributes to our understanding of the implications of the COVID-19 pandemic on CRC screening programs across Canada, there are several limitations. One limitation is the possibility of duplicate survey responses within the same P/T, which could have altered the accuracy of the conclusions. Additionally, not all representatives provided information for all the questions within the survey, resulting in missing data from specific regions. Furthermore, the study was limited by the lack of available and consistently reported data on the number of people awaiting colonoscopies in all P/Ts due to positive FITs or other reasons—which could have impacted the accuracy of the conclusions about the pandemic’s impact on CRC screening backlog. The lack of available data from various P/Ts about the exact number of missed CRC screening services/interruptions further limits the findings’ internal validity. However, P/Ts may have lacked data collection procedures to collect adequate data during the COVID-19 pandemic, so the long-term impact on CRC screening and treatments cannot be adequately predicted—requiring further research. In spite of these limitations, the revelations garnered from this study can serve as valuable inputs for policy formulation, enabling enhancements in the accessibility and robustness of CRC screening programs across Canada.

### 4.4. Recommendations

The study found several important insights that can help guide future efforts in responding to pandemic-like situations. We suggest the following recommendations based on the information provided by the representatives to mitigate the impact of COVID-19 on CRC screening programs: (1) Prioritizing high-risk patients for follow-up testing such as colonoscopy/endoscopy over average-risk individuals; (2) Increasing intersectoral collaboration and communication among multiple sectors, including HCPs, for better coordination of CRC screening services and reducing patient wait times; (3) Conducting awareness campaigns through screening invitations and health promotion campaigns to encourage the eligible screening population to participate in CRC screening; (4) Implementing measures to encourage individuals without a PCP or access to regular follow-up to participate in CRC screening by allowing other authorized HCPs to initiate screening; (5) Additional funding to increase the capacity of the CRC screening services; (6) Having a more efficient data collection system that allows for timely, comprehensive, and accurate data (e.g., monthly data about FIT and colonoscopies completed) to be accessible to researchers and policymakers. 

### 4.5. Conclusions

The COVID-19 pandemic has had a substantial impact on CRC screening programs in Canada, leading to the suspension of screening services and subsequent screening backlogs. Our study has unearthed several intervention measures that possess the potential to render CRC screening programs more accessible and resilient. Furthermore, the outcomes of the study underscore the imperative nature of readily accessible information and augmented resources within the healthcare system, which is instrumental in mitigating the repercussions of the pandemic on the screening process. Our investigation further revealed that effectively addressing the aftermath of the pandemic’s impact on CRC screening services mandates a multifaceted strategy, encompassing prioritization, collaborative efforts across sectors, enhanced communication, targeted awareness campaigns, financial backing, and the resolution of impending research challenges. Following these guidelines can assist in mitigating the impact of COVID-19 on CRC screening services and processes, as well as ensuring that patients receive timely and appropriate care. Further research is needed to address the study’s weaknesses and to continue to develop effective measures to lessen the pandemic’s effects on CRC screening programs.

## Figures and Tables

**Table 1:** Number of representatives per province/territory.

Province/Territory	Respondents
Alberta (AB)	3
British Columbia (BC)	1
Manitoba (MB)	1
New Brunswick	1
Newfoundland and Labrador (NL)	1
Nova Scotia (NS)	1
Ontario (ON)	5
Prince Edward Island (PEI)	1
Quebec (QC)	1
Saskatchewan (SK)	1
Northwest Territories (NWT)	2
Nunavut (NU)	2
Yukon (YT)	1

## Data Availability

The data presented in this study are available in this article.

## Appendix A

**Canada Landscape Assessment—Colorectal Cancer Care post COVID-19 Questionnaire**
**Purpose**

The COVID-19 pandemic introduced a multitude of challenges to the cancer care delivery system. Colorectal cancer prevention and diagnostic tests such as fecal immunochemical tests (FIT) and colonoscopies declined dramatically during the first wave of the pandemic. Since then, the cancer control communities across provinces have struggled to quantify the amount of missing screening, procedures and cancer diagnoses.

Colorectal Cancer Canada (CCC) is disseminating this survey to better understand the impact of these interruptions with the goal of finding solutions to mitigate the impact of missed screenings, increase screening and create greater resilience in cancer prevention and screening in Canada.

Please answer these questions to the best of your ability and knowledge.

1. “I agree that this information is being provided voluntarily and by providing this information I consent to its use by CCC for statistical purposes.”
☐Agree
☐ Disagree (please exit survey)
2. First and last name:
*3. Email address:
*To be contacted only if we need further clarification on any of your responses
4. Which province or territory do you represent?
☐Alberta
☐British Columbia
☐Manitoba
☐New Brunswick
☐Newfoundland and Labrador
☐Nova Scotia
☐Ontario Prince
☐Edward Island
☐Quebec
☐Saskatchewan
☐Northwest
☐Territories
☐Nunavut
☐Yukon

**Data on Screening Interruptions**

Please report “N/A” on any question to which you think data is not available.

5. Has colorectal cancer screening been interrupted in your province/territory between March 2020 and March 2022?
☐Yes
☐No
6. What is the approximate total number of days/weeks/months for which colorectal cancer screening was suspended in your province/territory? (between March 2020-March 2022)
7. (Follow-up question) If so, between what dates did these interruptions occur? example: March 2020–May 2020
8. Were there particular areas in your province/territory that were more impacted than others? (rural, urban or both)
9. Approximately how many individual colorectal cancer screenings have been missed in your province/territory? If this exact data is not available, can you report year-over-year changes (perhaps, monthly numbers compared with pre-pandemic numbers)? (March 2020–March 2022)
10. How many people are currently awaiting colonoscopies as a result of a positive FIT test? (March 2020–March 2022)
11. How many people are currently awaiting colonoscopies or endoscopies for any reason (including rectal bleeding)? (March 2020-March 2022)
12. What was the average delay for colonoscopies as a result of a positive FIT test? (March 2020-March 2022)

**Provincial/Territorial Plans to Address Backlog**

13. Does your province/territory have a plan to address the backlog of colorectal cancer screening with FIT tests? If so, what is it?
14. Does your province/territory have a plan to address the backlog of colorectal cancer screening with colonoscopies? If so, what is it?
15. (Follow-up question) How will this be addressed within the context of backlogs for all endoscopy, surgery (& other procedure) and a potential increase in later stage CRC due to suspended screening?
16. Will there be any changes to the screening program invitations to increase uptake? e.g., changes in kit distribution, navigators, integration with other screening programs?
17. In your opinion and based on your current plans, what is the anticipated amount of time that it would take for FIT and colonoscopies to reach pre-pandemic system performance? Or when (what date) do you expect to have returned to pre-pandemic performance?
18. Given the fact that there has been a 2-year hiatus, does your program plan to simply resume screening as before with the expectation of a higher positivity rate and a need for more colonoscopies?
19. In your opinion and based on your current plans, what is the anticipated amount of time it would take to catch up on overdue FIT tests and colonoscopies scheduled between March 2020 and March 2022?
20. In your opinion what do you think would have the greatest impact in avoiding future interruptions in colorectal cancer screening (specifically FIT)?
☐Increasing human resources in hospitals
☐Creating/Increasing out-of-hospital endoscopy clinics
☐Timely screening program data to inform capacity planning
☐Increasing endoscopy service hours
☐Making FIT tests more readily available
☐Increasing resources for community health centres to distribute FIT kits
☐Other (please specify)
21. In your opinion what do you think would have the greatest impact in avoiding future interruptions in follow-up colonoscopies?
☐Increasing human resources in hospitals
☐Creating/Increasing out-of-hospital endoscopy clinics
☐Timely screening program data to inform capacity planning
☐Increasing endoscopy service hours
☐Other (please specify)

**Importance of CRC Screening Data**

22. Would additional data have helped you mitigate the interruptions in FIT screening and endoscopy? Please explain.
23. What type of data (concerning screening and endoscopy) might have helped you mitigate the interruptions in FIT screening and endoscopy?
24. What other solutions do you suggest for addressing this issue?
25. Do you believe the lack of data collection on colorectal cancer screening, diagnostic, and other related procedures prevented you from answering these questions accurately?
☐ Yes
☐ No
☐Other (please specify)
